# Leaving Nothing Behind: Expanding the Clinical Frontiers of Drug-Coated Balloon Angioplasty in Coronary Artery Disease

**DOI:** 10.3390/jcdd12050176

**Published:** 2025-05-05

**Authors:** Marcello Marchetta, Stefano Sasso, Vincenzo Paragliola, Valerio Maffi, Gaetano Chiricolo, Gianluca Massaro, Giulio Russo, Daniela Benedetto, Saverio Muscoli, Giuseppe Colonna, Alessandro Mandurino-Mirizzi, Bernardo Cortese, Giuseppe Massimo Sangiorgi, Giuseppe Andò

**Affiliations:** 1 Department of Clinical and Experimental Medicine, University of Messina, 98122 Messina, Italy; marcello.marchetta1997@gmail.com; 2 Department of Biomedicine and Prevention, University of Rome “Tor Vergata”, 00133 Roma, Italy; stefanosasso95@libero.it (S.S.); paragliola.vincenzo@ptvonline.it (V.P.); maffi.valerio@gmail.com (V.M.); gaetano.chiricolo@ptvonline.it (G.C.); gianluca88massaro@gmail.com (G.M.); giuliorusso.md@gmail.com (G.R.); daniabenedetto@gmail.com (D.B.); saveriomuscoli@gmail.com (S.M.); gsangiorgi@gmail.com (G.M.S.); 3 Division of Cardiology, “V. Fazzi” Hospital, 73100 Lecce, Italy; giuseppe.colonna@tin.it (G.C.); ale.mandurinomirizzi@gmail.com (A.M.-M.); 4 Fondazione Ricerca e Innovazione Cardiovascolare, 20143 Milan, Italy; bcortese@gmail.com; 5 DCB Academy, 20143 Milan, Italy; 6 University Hospitals Harrington Heart and Vascular Institute, Cleveland, OH 44106, USA

**Keywords:** percutaneous coronary intervention, coronary artery disease, drug-coated balloons, drug-eluting stents, complex morphologies, coronary angioplasty, diabetes mellitus, high hemorrhagic risk, acute coronary syndromes

## Abstract

Drug-coated balloons (DCBs) have emerged as a promising alternative therapeutic strategy to traditional drug-eluting stent (DES) implantation in various coronary artery lesion scenarios, aiming to minimize complications associated with permanent metallic scaffolds, such as chronic inflammation, delayed vessel healing, and stent thrombosis. This review systematically evaluates the current clinical evidence supporting the use of DCBs across diverse anatomical and clinical contexts, including small-vessel disease, in-stent restenosis, bifurcation lesions, diffuse coronary lesions, acute coronary syndromes, and chronic total occlusions, as well as in special patient populations such as individuals with diabetes mellitus or at high bleeding risk. The literature analysis incorporated recent randomized controlled trials, observational studies, and real-world registries, highlighting the clinical efficacy, safety profiles, and specific advantages of DCB angioplasty. The findings consistently demonstrated non-inferior clinical outcomes of DCBs compared to DESs across multiple lesion types, with particular benefits observed in special populations, including reduced restenosis rates and comparable major adverse cardiac events (MACEs). Nevertheless, clinical data gaps remain, emphasizing the need for larger, longer-term randomized trials to refine patient selection and procedural techniques. In conclusion, DCB angioplasty represents a viable and effective alternative to conventional stenting, particularly advantageous in complex lesions and specific patient subsets, pending further definitive evidence.

## 1. Introduction and Rationale

Coronary artery disease (CAD) remains a primary cause of morbidity and mortality worldwide [1]. Over the past three decades, percutaneous coronary intervention (PCI) has become a cornerstone in managing CAD, initially through bare-metal stents (BMSs) and later refined by drug-eluting stents (DESs). While DESs substantially reduce in-stent restenosis (ISR) compared to BMSs, challenges persist—particularly in complex lesion subsets such as small vessels, long or diffuse disease, and bifurcation lesions [1]. Moreover, prolonged dual antiplatelet therapy (DAPT), often required after stent implantation, poses heightened bleeding risks for certain patients, motivating the search for alternative PCI strategies [2,3].

Drug-coated balloons (DCBs) have emerged as an important option in this context. Unlike stents, DCBs do not leave a permanent metallic scaffold; instead, they deliver a local, short-term burst of an antiproliferative drug (e.g., paclitaxel or sirolimus) upon balloon inflation [4]. This approach targets neointimal hyperplasia, seeking to preserve the vessel’s native anatomy—a concept widely referred to as “leave nothing behind” [5]. DCBs have become especially relevant for the following:

- ISR. Pioneering data from paclitaxel-coated balloon studies demonstrated that DCBs could effectively treat ISR without adding further stent layers [4,6].
- Small Coronary Arteries. Clinical trials have shown that in vessels <3 mm, drug-coated balloon angioplasty can be non-inferior to DESs, reducing the need for a permanent implant [7,8,9].
- Complex Lesion Subsets. Bifurcation side branches, long diffuse segments, and heavily calcified disease often pose increased technical and clinical risks for stenting. DCBs may simplify the procedure and reduce future reintervention complexity [1].
- High Bleeding Risk (HBR) Patients. Because no scaffold remains after a DCB-only PCI, the recommended DAPT duration may be shorter, which is advantageous for patients unable to tolerate extended antiplatelet regimens [10,11,12].

**Figure 1:**
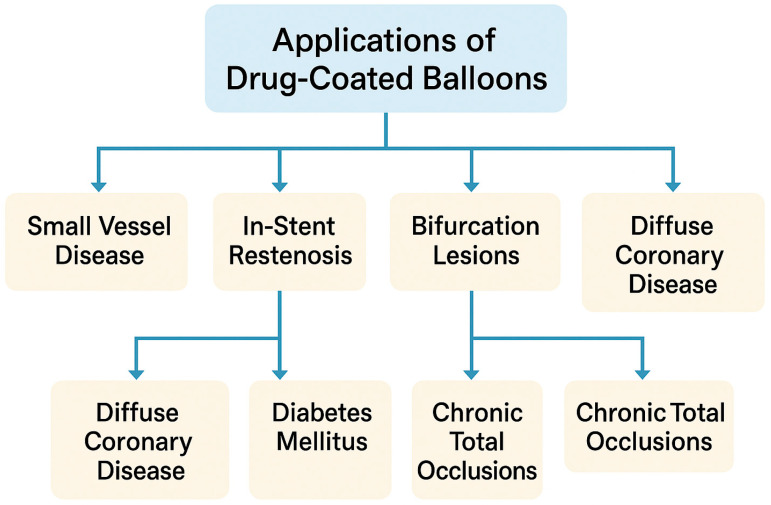
DCB angioplasty in coronary artery disease.

Over the last decade, multiple clinical trials and registries have confirmed the safety and efficacy of DCB-based PCI in a range of indications [4,6]. However, the adoption of DCBs varies geographically, influenced by local regulatory approvals, operator experience, and the availability of modern balloon technologies (including sirolimus- and novel paclitaxel-coated platforms). This review aims to provide a comprehensive, narrative overview of current DCB use across diverse lesion types (Figure 1), summarizing key clinical evidence, discussing patient selection, and highlighting the procedural aspects that may expand the role of DCBs as a “leave nothing behind” strategy.

## 2. Methods

To identify relevant studies, we conducted a structured literature search using the PubMed, Scopus, and Cochrane Library databases. The following search terms were used: (‘drug coated balloon’ OR ‘drug eluting balloon’ OR DCB OR DEB) AND (‘coronary artery disease’ OR ‘PCI’ OR ‘angioplasty’). We included randomized controlled trials, observational studies, and registry data published in English up to March 2025. Studies focusing on coronary applications of DCBs in various lesion subsets were selected. We excluded duplicate studies, case reports, non-coronary interventions, and articles without full text. Additional relevant articles were identified through manual reference screening.

## 3. Procedural Considerations for Optimal Drug-Coated Balloon Angioplasty

Achieving optimal outcomes with drug-coated balloon (DCB) angioplasty requires meticulous procedural technique. Adequate lesion preparation is fundamental to ensure uniform drug delivery and minimize complications such as recoil or dissection. Pre-dilatation using semi-compliant, non-compliant, or scoring balloons is recommended to modify plaque morphology and optimize vessel compliance. A balloon-to-artery ratio of 1:1 should be employed, and inflation time should be maintained for at least 30 to 60 s to maximize drug transfer to the vessel wall. After DCB deployment, a 5–10 min observation period before acquiring the final angiographic image is advisable to assess vessel recoil. Procedural success is generally defined as residual stenosis <50% with no flow-limiting dissections beyond type B. Operators must also avoid direct manipulation of the drug-coated segment to minimize drug loss during device handling and delivery. Notably, the therapeutic goal of DCB angioplasty is not to achieve an angiographic result equivalent to stenting, but rather to deliver an effective antiproliferative drug dose while preserving native vessel integrity and avoiding permanent implants.

## 4. Clinical Applications of Drug-Coated Balloons: Lesion-Specific Evidence

### 4.1. Small-Vessel Disease

PCI in small coronary arteries (often defined as vessels ≤ 2.75–3.0 mm in diameter) remains challenging due to increased risks of restenosis, driven mainly by limited luminal areas, suboptimal stent expansion, and subsequent neointimal hyperplasia. Despite advances in DES technology, outcomes in small-vessel interventions remain suboptimal, when compared with large-vessel interventions [7,13,14]. DCB angioplasty has emerged as an attractive alternative to address these limitations by delivering antiproliferative medication locally without permanent metal implantation, potentially reducing long-term inflammation and simplifying future revascularizations [15]. Initial clinical evidence supporting the efficacy of DCBs in small vessels was provided by the PEPCAD I trial, which demonstrated promising angiographic results with paclitaxel-coated balloons in vessels averaging 2.36 mm in diameter. At six months, this early trial reported a relatively low binary restenosis rate, validating the feasibility of a DCB-only strategy for these challenging lesions [6,16]. Subsequently, the BELLO trial compared paclitaxel-coated balloon angioplasty directly with paclitaxel-eluting stents in coronary arteries ≤2.8 mm. This randomized multicenter study found significantly lower late lumen loss with the paclitaxel-coated balloon (0.08 mm vs. 0.29 mm, *p* = 0.001) at six-month angiographic follow-up. Clinical endpoints at one year—including target lesion revascularization (TLR) and major adverse cardiac events (MACEs)—were comparable between the two approaches, reinforcing the rationale of avoiding permanent stents in small coronary arteries [17]. The pivotal BASKET-SMALL 2 trial provided further definitive evidence by randomizing patients with de novo small-vessel lesions (<3.0 mm) to paclitaxel-coated balloons or second-generation DESs. At a one-year follow-up, the DCB approach showed non-inferiority compared to DESs, with similar MACE rates (7.3% vs. 7.5%; hazard ratio 0.97, 95% CI 0.58–1.64, *p* = 0.92). This trial, with its robust design and adequate power, significantly influenced clinical practice by establishing DCBs as an acceptable and effective alternative to DESs in small vessels [15,18]. The RESTORE SVD China trial extended these findings to an Asian population, enrolling patients with coronary vessels between 2.25 mm and 2.75 mm. At a nine-month angiographic follow-up, in-segment diameter stenosis was non-inferior in the paclitaxel-coated balloon group compared to DESs (29.6% vs. 24.1%, *p* for non-inferiority = 0.01). Clinical outcomes at one year were also comparable, adding to the robustness of evidence supporting DCB use in small vessels across different populations [19]. Finally, the recent PICCOLETO II trial compared paclitaxel-coated balloons with everolimus-eluting stents in vessels ≤ 2.75 mm. At a three-year clinical follow-up, DCB therapy showed numerically lower rates of TLR and comparable clinical outcomes, confirming the long-term safety and effectiveness of the latest-generation paclitaxel-coated balloons in treating small coronary arteries [8]. Taken together (Table 1) and consistently with a recent individual patient data meta-analysis [9], these studies demonstrate that in selected patients with small-vessel coronary artery disease, DCB angioplasty provides clinical outcomes comparable to DESs, while offering additional benefits such as the avoidance of permanent implants, reduced complexity in future reinterventions, and potentially shorter durations of DAPT.

### 4.2. In-Stent Restenosis

Despite significant advances in DES technology, ISR continues to be a challenging clinical entity, often requiring repeat interventions and complex therapeutic approaches. Traditionally, ISR was managed with additional stent implantation, which could result in multiple overlapping metallic layers, exacerbating chronic inflammation and potentially compromising future interventions. DCB angioplasty has emerged as an attractive stent-free alternative, providing localized antiproliferative drug delivery to treat ISR effectively while avoiding further implantation of permanent scaffolds [20]. Initial robust evidence demonstrating the effectiveness of DCB angioplasty in ISR management came from the landmark PEPCAD II trial. This randomized study included patients with ISR lesions previously treated with bare-metal stents and showed that paclitaxel-coated balloons significantly reduced in-segment late lumen loss (0.03 mm vs. 0.74 mm, *p* < 0.001) and lowered the incidence of MACEs compared to conventional balloon angioplasty at six months, providing early, compelling clinical justification for a DCB approach in ISR patients [4]. The subsequent PEPCAD-DES trial expanded these findings specifically to DES-related ISR. Patients randomized to paclitaxel-coated balloon therapy exhibited significantly reduced late lumen loss at a six-month angiographic follow-up compared with plain balloon angioplasty (0.43 mm vs. 1.03 mm, *p* < 0.001), with correspondingly lower rates of binary restenosis (17.2% vs. 58.1%, *p* < 0.001) and target lesion revascularization (TLR; 15.3% vs. 36.8%, *p* = 0.005), solidifying DCBs as an effective strategy in DES-associated ISR [6]. Confirming and extending these findings, the ISAR-DESIRE 3 trial directly compared paclitaxel-coated balloons to paclitaxel-eluting stents (PESs) for treating DES restenosis. At a one-year follow-up, both approaches had similar clinical outcomes, with the paclitaxel-coated balloon meeting criteria for non-inferiority compared to PESs regarding the primary composite endpoint (13.5% DCB vs. 14.0% PES, *p* = 0.007 for non-inferiority). This trial supported the role of DCBs as a valid alternative to repeat stenting in DES-related ISR [21]. Similarly, the RIBS IV trial compared paclitaxel-coated balloon angioplasty with everolimus-eluting stents (EESs). Although EESs showed superior angiographic results and lower rates of TLR at one year, the paclitaxel-coated balloon demonstrated acceptable clinical efficacy and was endorsed particularly for cases where avoiding additional stent layers was clinically beneficial (freedom from TLR at 1 year: 90% EES vs. 81% DCB, *p* = 0.02) [22]. Expanding the evidence base in Asian populations, the PEPCAD China ISR trial confirmed that paclitaxel-coated balloon angioplasty provided non-inferior late lumen loss at a nine-month angiographic follow-up compared with repeat paclitaxel-eluting stenting (0.46 mm vs. 0.55 mm, *p* < 0.001 for non-inferiority), demonstrating the consistency of the DCB strategy across different geographic populations [23]. More recently, the AGENT IDE trial provided definitive evidence for DCB superiority over plain balloon angioplasty in a large cohort of U.S. patients with ISR. At a 12-month clinical follow-up, DCB therapy significantly reduced target lesion failure compared with standard balloon angioplasty (17.9% vs. 28.7%; hazard ratio, 0.58; 95% CI, 0.41–0.83; *p* = 0.003), firmly establishing DCB angioplasty as the treatment of choice for DES-ISR in routine clinical practice [24]. Novel balloon platforms have also been tested, for example, in the SABRE trial, where a sirolimus-coated balloon showed promising results, achieving low six-month late lumen loss (0.31 mm) and acceptable one-year clinical outcomes (TLR 14.3%). These findings suggest expanding potential treatment options beyond paclitaxel-based devices [25]. A head-to-head comparison of antiproliferative agents was addressed by Scheller et al. [26], who conducted a combined analysis of two randomized trials comparing sirolimus-coated and paclitaxel-coated balloons for DES-ISR. Both technologies yielded very similar late lumen loss at a six-month follow-up (0.25 mm sirolimus vs. 0.27 mm paclitaxel, *p* = 0.94), demonstrating comparable efficacy between these drug types for ISR management [27]. Finally, the BIOLUX trial investigated a novel paclitaxel-coated balloon formulation (BTHC excipient), showing favorable angiographic and clinical results at a nine-month follow-up with low rates of binary restenosis (13.6%) and TLR (6.8%) [28]. Recent registry data support the superiority of ultrathin-strut DESs over DEBs in patients with diffuse in-stent restenosis. In a multicenter study including 1367 patients, ultrathin DESs were associated with a significant reduction in TLR, TVR, and DOCE compared to DEBs, particularly in diffuse ISR subsets, while outcomes were similar for focal ISR [29]. These outcomes underscore ongoing innovations in balloon technology, supporting the robustness and continuous evolution of the DCB approach for ISR [28]. Taken together (Table 1), these extensive randomized clinical data clearly demonstrate the established clinical value of DCB angioplasty as a highly effective and guideline-endorsed therapy for ISR, offering advantages such as avoiding additional permanent implants, reducing long-term vessel complications, and facilitating future treatment strategies [30].

### 4.3. Bifurcation Lesions

Coronary bifurcation lesions continue to represent a major technical challenge in interventional cardiology due to their inherent complexity and the increased risk of restenosis and adverse clinical outcomes. The optimal treatment strategy remains debated, with uncertainties around the most effective use of stent-based versus non-stent-based techniques, particularly for side branches [31]. In this context, drug-coated balloons (DCBs) offer a potentially valuable strategy by reducing the implantation of permanent metallic scaffolds, thus minimizing long-term inflammation and restenosis and preserving future interventional options. The clinical utility of DCB angioplasty in bifurcation lesions has been evaluated in several key studies. The multicenter, randomized PEPCAD-BIF trial assessed the effectiveness of paclitaxel-coated balloons compared with conventional balloon angioplasty for bifurcation side branches following main-branch stenting. At a nine-month angiographic follow-up, the DCB group demonstrated significantly reduced late lumen loss compared to conventional balloon angioplasty (0.13 ± 0.51 mm vs. 0.51 ± 0.48 mm; *p* = 0.013), although TLR rates were similar between the groups (9.4% vs. 10.3%, respectively; *p* = 0.96). The results highlighted DCBs as a safe and effective adjunctive treatment in the side branches of bifurcation lesions, potentially reducing angiographic restenosis and simplifying the procedural strategy [32]. Further support for a DCB approach in bifurcation lesions was provided by the DEBSIDE trial. In this prospective observational study, patients with coronary bifurcation lesions involving side branches were treated with a paclitaxel-coated balloon following main-branch stenting. At 12 months, the use of DCBs was associated with a low binary restenosis rate (7.7%) and acceptable clinical outcomes, demonstrating the feasibility and clinical benefit of the DCB approach for side-branch treatment in bifurcation PCI. The DEBSIDE trial reinforced the clinical rationale for minimizing stent implantation in bifurcation lesions by relying on drug delivery without permanent scaffolds [33]. The concept of combining DCBs with stent implantation—known as the hybrid strategy—has also been explored. The HYPER study examined procedural and clinical outcomes following hybrid PCI, involving main-branch DES implantation and side-branch DCB angioplasty. The study demonstrated favorable clinical outcomes at a one-year follow-up, with low rates of target lesion revascularization (5.5%) and major adverse cardiovascular events (MACEs, 8.8%), thus supporting that the hybrid approach is clinically beneficial for complex bifurcation lesions. This strategy optimally exploits the advantages of both DES and DCB technologies, offering an effective alternative to the more extensive two-stent techniques traditionally employed in bifurcation PCI [34]. Moreover, the more recent EASTBOURNE-BIF registry assessed the clinical outcomes of DCB-only strategies for both main and side branches in coronary bifurcation lesions. In this real-world registry, DCB-only angioplasty yielded encouraging clinical results at 12 months, with low rates of TLR (4.7%) and MACEs (7.5%), thereby confirming the practicality and clinical utility of a “leave nothing behind” approach even in complex bifurcation settings. The EASTBOURNE registry underscored the growing clinical acceptance of stent-free interventions and provided further support for the broader adoption of DCB therapy as a routine treatment modality for bifurcation lesions. Overall, these data (Table 1) collectively demonstrate that DCB angioplasty, either alone or as part of a hybrid PCI strategy, provides effective management of coronary bifurcation lesions. The avoidance or minimization of permanent scaffolds appears particularly advantageous, limiting chronic inflammatory responses, preserving vessel geometry, and facilitating future interventions. Therefore, DCB-based approaches deserve consideration as valid therapeutic options in carefully selected bifurcation lesion scenarios, especially when side-branch preservation and reduced complexity in future revascularizations are desirable clinical objectives.

### 4.4. Diffuse Coronary Artery Disease

Diffuse coronary artery disease, defined by lesions generally exceeding 25 mm, remains a significant challenge in interventional cardiology. Treatment traditionally involves multiple overlapping DESs, which are associated with increased risks of restenosis, stent fracture, and thrombosis due to prolonged vessel inflammation and complex stent interactions [35,36]. DCB angioplasty, alone or in a hybrid strategy combining DCBs and DESs, offers a potential alternative by reducing metal implantation, thus potentially lowering long-term adverse outcomes and preserving treatment options for future interventions [37]. Early data supporting DCB use in diffuse coronary lesions emerged from an observational study by Costopoulos et al. [38]. Their retrospective analysis compared clinical outcomes of diffuse lesions (>25 mm) treated with a DES alone or with a hybrid DES plus DCB approach. At two years, the hybrid approach demonstrated similar clinical outcomes to the DES-only strategy, with comparable major adverse cardiac event (MACE) and target lesion revascularization (TLR) rates, while significantly reducing overall stent length (24.8 mm vs. 41.9 mm, *p* < 0.001). This finding provided early evidence that limiting metal implantation in diffuse lesions was feasible without compromising clinical effectiveness [38]. Further evidence came from the SPARTAN DCB study, evaluating the long-term safety of standalone paclitaxel-coated balloon angioplasty in extensive de novo lesions (mean lesion length 26.05 ± 11.95 mm). With a median follow-up of nearly four years, the DCB-alone strategy showed favorable long-term survival and low rates of TLR comparable to contemporary DESs. These results supported the clinical viability and durability of DCB therapy as a stand-alone strategy for managing diffuse coronary disease [39]. A retrospective study by Leone et al. [40] further expanded these insights, focusing on diffuse coronary lesions in larger vessels (≥3.0 mm diameter, mean lesion length 45 ± 26 mm). Their DCB-only strategy achieved high procedural success (97.8%) and a low TLR rate of 5.8% at a 12-month follow-up [40]. These outcomes emphasized that DCB therapy could effectively manage extensive lesions even in larger coronary arteries, confirming its broad clinical applicability. Hybrid strategies combining a DES proximally and a DCB distally have also been successfully applied. Xu et al. [41] retrospectively assessed this hybrid approach in diffuse lesions, achieving excellent procedural success (97.2%) and a low TLR rate (5.5%) at a 19-month median follow-up. The study highlighted that strategically limiting stent implantation to proximal segments, while treating distal segments with DCBs, could optimize clinical outcomes by minimizing overall stent length [41]. Finally, the prospective multicenter HYPER study provided contemporary evidence supporting a hybrid DES plus DCB approach. This strategy yielded excellent one-year clinical outcomes (TLR 5.5%, MACEs 8.8%), further reinforcing the clinical benefit and safety of integrating DCB technology with targeted stenting in the treatment of complex, diffuse coronary lesions [34]. Taken together, these studies (Table 1) provide compelling evidence supporting DCB angioplasty, either as a standalone treatment or as part of a hybrid strategy, as an effective therapeutic option for diffuse coronary artery disease. By significantly reducing or avoiding permanent metal implantation, DCB-based strategies appear advantageous in managing these challenging lesions, potentially minimizing chronic vessel inflammation, reducing long-term complications, and preserving options for future revascularizations.

### 4.5. Chronic Total Occlusions (CTOs)

Chronic total occlusions (CTOs) remain among the most challenging coronary lesions encountered in interventional cardiology. Despite technological advances, procedural success rates remain suboptimal, and restenosis following successful recanalization continues to be a clinical concern [42]. DCBs represent a potential strategy for addressing restenosis after CTO intervention, offering targeted antiproliferative treatment without the drawbacks associated with permanent metallic scaffolding. Initial observational data on the efficacy of DCBs in CTO interventions were provided by Köln et al. [43], who evaluated the outcomes of paclitaxel-coated balloon angioplasty following successful CTO recanalization. In their retrospective analysis of 34 consecutive patients, DCB treatment demonstrated a primary angiographic success rate of 100%, with encouraging clinical outcomes at 12 months, including a low TLR rate of 8.8% and a MACE rate of 11.8% [43]. These findings suggested that DCB angioplasty is safe and effective in maintaining vessel patency after CTO recanalization, providing a viable alternative to routine DES implantation. Further robust clinical evidence emerged from the study by Wang et al. [44], comparing DCBs with second-generation DESs in patients undergoing successful recanalization of CTOs. At up to 3 years of follow-up, both strategies demonstrated comparable safety and efficacy outcomes. There were no significant differences in LLL, TLR, or MACEs [44]. This trial confirmed the non-inferiority of DCBs compared to DESs in maintaining long-term patency after CTO recanalization, indicating that a stent-free approach could effectively minimize restenosis without compromising clinical outcomes. A recent systematic review and meta-analysis [45] including 10 studies and 1,695 patients evaluated the use of DCBs in the treatment of CTOs. The analysis demonstrated that DCBs yielded consistent late luminal gains and minimal lumen loss at 7–12 months, with no significant differences in TLR or MACEs when compared with DESs or hybrid strategies (DES + DCB) in both de novo and in-stent CTO populations [45]. These findings support the safety and potential efficacy of DCBs as an alternative revascularization strategy for CTOs, although randomized controlled trials are warranted to further validate these results. Overall, available evidence (Table 1) suggests that DCB angioplasty is a safe and effective treatment modality after successful CTO recanalization. The absence of permanent metallic implants potentially reduces chronic inflammation and simplifies future revascularization attempts, making DCBs a particularly appealing option in patients undergoing complex CTO procedures. Nonetheless, additional randomized controlled trials with larger sample sizes and longer follow-up durations are warranted to further consolidate the role of DCBs in routine CTO management.

**Table 1:** Lesion-specific evidence for DCB angioplasty.

Lesion Type	Study	Design	No. of Patients	Primary Endpoint	Follow-Up	Main Findings
**Small-vessel disease**	Basket Small-2 [15,18]	RCT	758	MACEs (CV death, MI, TVR)	12 months	DCB non-inferior to DES (7.5% vs. 7.3%, *p* = 0.918)
	Piccoleto Ii [8]	RCT	232	LLL, MACEs	12 months	Superior to DES (0.04 vs. 0.17 mm, *p* = 0.03)
	Restore Svd China [19]	RCT	230	Diameter stenosis, TLF	24 months	DCB and DES comparable
**In-stent restenosis**	Ribs Iv [22]	RCT	309	In-segment LLL, TLR, MACEs	36 months	Superior to POBA, comparable to DES
	Isar-Desire 3 [21]	RCT	402	Diameter stenosis (%), TLR, MI	36 months	Comparable to DES, superior to POBA
**Bifurcation lesions**	Pepcad-Bif [32]	RCT	64	Angiographic LLL, TLR	9 months	DCB superior vs. POBA (0.13 vs. 0.51 mm, *p* = 0.013)
	Debside [33]	Observational	52	TLR	12 months	Effective side-branch treatment, TLR 7.7%
	Hyper [34]	Observational	210	MACEs (CV death, MI), TLR	12 months	Hybrid strategy effective, low TLR (5.5%)
**Diffuse coronary lesions**	Spartan [39]	Observational	1517	All-cause mortality	NR	Similar mortality to DES
	Leone et al. [40]	Observational	93	MACEs (CV death, MI), TLR	12 months	High procedural success, low TLR (5.8%)
	Costopoulos et al. [38]	Observational	212	MACEs (CV death, MI), TLR	12 months	Outcomes comparable to DES, less stenting
	Xu et al. [41]	Observational	109	MACEs, TLR	12 months	Hybrid approach effective, low TLR
**Chronic total occlusions**	Koln et al. [43]	Observational	34	MACEs (CV death, MI), TLR	9 months	Safe, low MACE rate (11.8%), feasible
	Wang et al. [44]	RCT	591	Angiographic LLL, MACEs	3 years	Less LLL in DCB, comparable restenosis and MACEs

DCB: drug-coated balloon; DES: drug-eluting stent; LLL: late lumen loss; MACE: major adverse cardiac event; TLR: target lesion revascularization; TVR: target vessel revascularization; RCT: randomized controlled trial.

## 5. Special Populations and Clinical Settings

### 5.1. High Bleeding Risk (HBR)

Patients at HBR undergoing PCI represent a particularly challenging subset due to their vulnerability to both thrombotic and hemorrhagic complications [46]. Historically, BMSs were preferred in these patients, primarily because of the shorter DAPT requirement compared to DESs. However, the increased incidence of restenosis associated with BMS implantation poses significant limitations to the widespread use of BMSs, prompting exploration of alternative strategies such as DCB angioplasty, either alone or combined with limited stenting. The combination of a BMS and DCB was assessed in the prospective, multicenter, non-randomized PANELUX trial [12]. In this study, patients with HBR were treated with a cobalt–chromium BMS (PRO-Kinetic Energy stent) followed by post-dilation with a paclitaxel-coated balloon (Pantera Lux). The primary endpoint, TLF, occurred in 5.6% of patients at 12 months. Clinically driven target lesion revascularization (cd-TLR) was notably low (2.9%), and despite the abbreviated DAPT duration (median 33 days), bleeding complications were acceptable, with a 7.2% incidence predominantly classified as moderate–severe (BARC 3) bleeding. Subgroup analyses revealed significantly increased TLF and bleeding rates in patients aged ≥80 years and those with renal insufficiency, underscoring the need for careful patient selection in this population [12]. Further evidence supporting DCB use in HBR patients comes from the DEBUT trial, a randomized study comparing DCB-only PCI with BMS implantation [11]. This trial clearly demonstrated the superiority of a DCB-only strategy over BMS, showing significantly reduced rates of major adverse cardiac events (MACEs; 1% DCB vs. 14% BMS, *p* < 0.0001) and target lesion revascularization (0% DCB vs. 6% BMS, *p* = 0.0012) at 12 months. Importantly, despite the shortened DAPT duration, bleeding rates were similar between the two groups, validating the safety of the DCB-only approach in HBR patients [11]. Registry-based evidence from Uskela et al. [47] further highlighted the safety and efficacy of DCBs in HBR patients. This study evaluated DCB-only angioplasty in a real-world population presenting with stable CAD or acute coronary syndrome (ACS). The DCB-only strategy achieved excellent clinical outcomes at 12 months, with low ischemia-driven TLR rates (1.4% in stable CAD, 2.8% in ACS) and a manageable significant bleeding rate (5.9%), despite the high prevalence of bleeding risk factors in the study population [47]. Similarly, a retrospective registry by Räsänen et al. [10] examined a DCB-only PCI strategy combined with single antiplatelet therapy (SAPT) after discharge in an elderly cohort at very high bleeding risk. This approach resulted in notably low TLR rates (0% in stable CAD, 3.0% in ACS) and acceptable bleeding complications (10.5% BARC 2–5 bleeding), even without a routine DAPT post-procedure. This study illustrated the feasibility and safety of a DCB-only approach in elderly, high-risk patients who cannot tolerate prolonged antithrombotic therapies [10]. Finally, the recent randomized ULTIMATE III trial [48] compared the outcomes of intravascular ultrasound (IVUS)-guided versus angiography-guided paclitaxel-coated balloon angioplasty in HBR patients. IVUS-guided DCB angioplasty demonstrated significantly reduced late lumen loss (0.14 ± 0.37 mm IVUS vs. 0.37 ± 0.58 mm angiography, *p* < 0.001) and improved minimal lumen diameters at a 7-month angiographic follow-up, suggesting that imaging-guided optimization may further enhance the clinical effectiveness of DCB interventions in high-risk, complex lesion subsets typical of HBR patients [48]. In conclusion, the current evidence (Table 2) supports DCB angioplasty, either as a stand-alone treatment or combined with limited stenting, as a safe and effective alternative to traditional stent implantation in HBR populations. Such strategies reduce the necessity for prolonged DAPT, consequently decreasing bleeding risk without compromising long-term clinical efficacy. The incorporation of advanced imaging modalities, such as IVUS, may further refine these treatment strategies, optimizing procedural outcomes and minimizing complications in this vulnerable patient population.

### 5.2. Acute Coronary Syndromes (ACSs)

ACSs encompass a spectrum of clinical conditions characterized by acute myocardial ischemia, ranging from unstable angina (UA) and non-ST-elevation myocardial infarction (NSTEMI) to ST-elevation myocardial infarction (STEMI). PCI with a DES remains the standard therapeutic approach [49]; however, concerns regarding delayed vessel healing, stent thrombosis, and restenosis persist, prompting an investigation into alternative strategies such as drug-coated balloons (DCBs). Initial evidence supporting the potential role of drug-coated balloon angioplasty in STEMI patients came from the DEB-AMI trial [50]. This randomized study compared outcomes among STEMI patients treated with a combination of a DCB and BMS, a BMS alone, or a DES. At six months, angiographic LLL was significantly lower in patients treated with a DES compared with a DCB + BMS and a BMS alone (0.09 mm DES vs. 0.49 mm DCB + BMS vs. 0.74 mm BMS; *p* < 0.001). However, despite angiographic superiority, clinical outcomes such as MACE rates were comparable between the DES and DCB + BMS (10.0% DES vs. 11.8% DCB + BMS), suggesting that DCB-based strategies may still provide clinical benefits, particularly when DESs are less desirable [50]. Subsequently, the PEPCAD NSTEMI trial evaluated the use of paclitaxel-coated balloon angioplasty compared to DES implantation in patients presenting with NSTEMI [51]. In this randomized multicenter trial involving 210 patients, DCBs demonstrated non-inferiority regarding the primary endpoint of late lumen loss at a nine-month follow-up compared to DESs (0.24 mm vs. 0.14 mm; *p* for non-inferiority <0.001). Clinical outcomes at 12 months, including MACEs, were also comparable (7.6% DCB vs. 7.2% DES, *p* = 0.89), highlighting the safety and efficacy of a stent-free PCI approach in NSTEMI patients [51]. Further evidence supporting DCB use in acute coronary settings came from Besic et al. [52], who compared the combination of a DCB plus a BMS versus a BMS alone in patients presenting with NSTEMI or unstable angina. Despite similar binary restenosis rates at a nine-month angiographic follow-up, there was a trend towards reduced cd-TLR rates in patients treated with the combination of a DCB and BMS compared to a BMS alone (4.5% vs. 7.9%, *p* = 0.29). These findings suggest that DCBs might reduce clinically relevant restenosis in acute coronary syndrome scenarios, although statistical significance was not reached due to limited study power [52]. The DEB-AMI trial results were complemented by real-world observational evidence from Vos et al. [53], who assessed outcomes following DCB angioplasty in STEMI. The study documented acceptable procedural success rates (97%) and low TLR rates (5.7%) at 12 months. These results reinforce the feasibility and practical safety of adopting DCB angioplasty in the STEMI population, especially in situations where avoiding additional metallic scaffolds may be beneficial [53]. Most recently, Merinopoulos et al. [54] analyzed the outcomes of a large registry comparing DCB-only angioplasty with DES implantation in STEMI patients. At a three-year follow-up, the DCB-only strategy showed comparable clinical outcomes to DESs, with no significant differences in cardiac death, myocardial infarction, or TLR (TLR rates: 7.0% DCB vs. 6.8% DES; *p* = 0.88). This real-world registry highlights the longer-term reliability of DCB angioplasty, even in STEMI, traditionally treated aggressively with DESs [54]. In conclusion, current evidence (Table 2) supports the clinical efficacy and safety of DCB-based approaches in acute coronary syndrome settings, including NSTEMI and STEMI. Despite the persistent angiographic advantages of DESs, DCBs represent a valid alternative, particularly advantageous in selected subsets where minimizing metallic implants is desirable. Future larger randomized studies are needed to refine patient selection criteria and further clarify the clinical positioning of DCBs in ACS management.

### 5.3. Diabetes Mellitus

Patients with diabetes mellitus (DM) undergoing PCI represent a subgroup at increased risk of adverse clinical events, including higher rates of restenosis, stent thrombosis, and recurrent ischemic complications. DCBs have emerged as a promising therapeutic option for diabetic patients, offering effective antiproliferative drug delivery while avoiding permanent metallic scaffolds, thereby potentially reducing late complications linked to persistent vessel inflammation [55]. A diabetic subgroup analysis of the BASKET-SMALL 2 trial evaluated paclitaxel-coated balloons versus second-generation DESs in small coronary vessels. At a one-year follow-up, DCBs demonstrated comparable clinical outcomes to DESs, with similar rates of major adverse cardiac events (MACEs: 10.1% DCB vs. 13.7% DES, HR 0.74, 95% CI 0.34–1.58, *p* = 0.52) [56]. This highlighted the clinical non-inferiority of DCB therapy even in the high-risk diabetic population with small-vessel coronary disease. Further supportive evidence emerged from the diabetic subanalysis of the PICCOLETO II trial, comparing paclitaxel-coated balloons with everolimus-eluting stents (EESs) in diabetic patients with small coronary vessels. At three-year follow-up, diabetic patients treated with DCBs exhibited favorable outcomes, including numerically lower target lesion revascularization (TLR) rates compared to EESs (7.5% DCB vs. 10.0% EES, *p* = NS) [57]. This confirmed the sustained effectiveness and safety of the DCB approach over an extended follow-up period in diabetic patients. The diabetic subgroup analysis from the RESTORE SVD China trial provided additional validation by comparing paclitaxel-coated balloons with drug-eluting stents specifically in diabetic patients. At a nine-month angiographic follow-up, late lumen loss (0.34 mm DCB vs. 0.30 mm DES, *p* = NS) and clinical outcomes at one year were comparable between groups. These findings reaffirmed the efficacy of DCBs as a reliable therapeutic alternative in diabetic patients with small coronary vessel disease [19]. In a real-world setting, the diabetic subgroup analysis from the EASTBOURNE registry further demonstrated the effectiveness and safety of paclitaxel-coated balloons. At a one-year follow-up, diabetic patients showed excellent clinical outcomes with low TLR (3.6%) and favorable overall MACE rates (6.5%), suggesting that the DCB-only strategy effectively managed diffuse coronary lesions, which are notoriously challenging in diabetic populations [58]. In the DM-Dragon registry, which focused specifically on diabetic patients, clinical outcomes were similar between DEBs and DESs. Notably, a significantly lower all-cause mortality was observed in the DEB arm (2.78% vs. 11.11%; *p* = 0.048), supporting the potential value of DEBs in selected diabetic subgroups [59]. Lastly, the EASTBOURNE-BIF registry specifically examined diabetic patients undergoing DCB angioplasty in coronary bifurcation lesions. The diabetic subgroup reported similarly favorable one-year outcomes, with low TLR (4.5%) and MACE rates (8.7%), further highlighting the versatility and clinical utility of DCB therapy in complex bifurcation settings among diabetic patients [60]. Collectively, current evidence (Table 2) demonstrates the clinical effectiveness and safety of drug-coated balloon angioplasty in diabetic patients across various lesion subtypes, including small-vessel disease, diffuse coronary lesions, and complex bifurcations. DCB therapy thus emerges as a beneficial alternative strategy, providing durable clinical outcomes, avoiding prolonged exposure to metallic implants, and potentially reducing inflammation-related complications inherent to diabetic coronary artery disease.

**Table 2:** Special populations and clinical settings.

Clinical Setting	Study	Design	No. of Patients	Primary Endpoint	Follow-Up	Main Findings
**High bleeding risk**	Debut [11]	RCT	208	MACEs (CV death, MI, TLR)	12 months	DCB superior to BMS (1% vs. 14%, *p* < 0.001)
	Panelux [12]	Observational	432	TLF, CV death, MI, TLR	12 months	Low TLF (5.6%), safe short DAPT duration (median 33 days)
	Ultimate Iii [48]	RCT	448	In-segment LLL	7 month	IVUS-guided DCB superior to angiography-guided
	Uskela et al. [47]	Observational	301	MACEs, BARC 2–5 bleeding	12 months	Low TLR (1.4–2.8%), acceptable bleeding rate (5.9%)
	Rasanen et al. [10]	Observational	114	TLR, bleeding	12 months	Low TLR (0–3%), acceptable bleeding with SAPT (10.5%)
**Diabetes mellitus**	BASKET-SMALL 2 (DM Substudy) [56]	RCT (subanalysis)	263	MACEs (CV death, MI, TLR)	12 months	DCB comparable vs. DES (10.1% vs. 13.7%, *p* = 0.52)
	PICCOLETO II (DM Substudy) [57]	RCT (subanalysis)	232	MACEs, TLR	36 month	Low TLR rates similar to DES, sustained benefit
	RESTORE SVD China (DM Substudy) [19]	RCT (subanalysis)	230	In-segment LLL, TLR	9 months	Comparable outcomes for DCB vs. DES (12% vs. 11.1%)
	EASTBOURNE (DM Substudy) [58]	Observational	424	MACEs, TLR	12 months	Low TLR (3.6%), favorable MACE (6.5%)
	EASTBOURNE-BIF (DM Substudy) [60]	Observational	210	MACEs, TLR	12 months	Effective in bifurcation lesions, low TLR (4.5%)
**Acute Coronary Syndrome (NSTEMI)**	Pepcad Nstemi [51]	RCT	210	LLL	9 months	DCB non-inferior to DES
	Besic et al. [52]	Observational	120	Restenosis rate, TLR	12 months	Lower TLR (4.5% DCB + BMS vs. 7.9% BMS alone, *p* = 0.29)
**Acute Coronary syndrome (STEMI)**	Deb-Ami [50]	RCT	150	LLL	6 months	DES superior angiographically, similar clinical outcomes
	Pappa [53]	Observational	100	MACEs (CV death, MI)	6 months	Feasible DCB-only STEMI, low MACE rate (5%)
	Merinopoulos et al. [54]	Observational	1139	TLR, MACEs	12 months	Comparable TLR and MACEs for DCB vs. DES (7.0% vs. 6.8%)

BMS: bare-metal stent; DAPT: dual antiplatelet therapy; DCB: drug-coated balloon; DES: drug-eluting stent; DM: diabetes mellitus; IVUS: intravascular ultrasound; LLL: late lumen loss; MACE: major adverse cardiac event; NSTEMI: non-ST elevation myocardial infarction; RCT: randomized controlled trial; SAPT: single antiplatelet therapy; STEMI: ST-elevation myocardial infarction; TLF: target vessel failure; TLR: target lesion revascularization.

## 6. Future Perspectives and Conclusions

DCB angioplasty has progressively emerged as a reliable and effective therapeutic alternative across various coronary lesion subsets, demonstrating encouraging outcomes in small-vessel disease, in-stent restenosis, bifurcations, diffuse coronary lesions, patients with high bleeding risk, diabetes mellitus, and CTOs. The clinical benefits primarily derive from the avoidance of permanent metallic implants, reduction in chronic vessel inflammation, and potential shortening of DAPT durations. Despite these advantages, several critical knowledge gaps remain. Most available evidence supporting the use of DCBs derives from observational studies, small randomized controlled trials, or subgroup analyses. Thus, adequately powered, large-scale randomized trials and robust registries with longer-term clinical follow-up remain necessary to establish definitive clinical guidelines and further validate the widespread use of DCBs. Future research should specifically focus on direct comparisons of DCB angioplasty versus newer-generation DESs, especially for complex lesion subsets like bifurcation lesions, diffuse coronary disease, and CTOs, where clinical outcomes remain uncertain. Moreover, emerging technologies, including sirolimus-coated balloons and novel excipients, represent promising areas for future development. Further comparative research investigating different antiproliferative drugs and coating platforms is required to clarify if clinical outcomes differ significantly between available DCB technologies. While paclitaxel-coated balloons (PCBs) remain the most widely studied and utilized devices, recent interest has focused on sirolimus-coated balloons (SCBs) as a potential alternative. SCBs differ significantly in pharmacokinetics and delivery platforms, using crystalline or microreservoir-based coatings that require more complex manufacturing and deployment techniques. Compared to paclitaxel, sirolimus has a broader therapeutic index and cytostatic rather than cytotoxic properties, which may result in improved vascular healing and a reduced inflammatory response. However, clinical experience with SCBs remains more limited, and most available data derive from single-arm registries or underpowered trials. Emerging studies have shown promising results, but randomized head-to-head comparisons with PCBs are still lacking. Additionally, due to the hydrophilic nature and higher molecular weight of sirolimus, SCBs may present challenges in ensuring uniform and deep vessel wall penetration, especially in calcified or complex lesions. Therefore, while SCBs represent a promising technological evolution, further comparative trials are warranted to establish whether their theoretical advantages translate into superior clinical outcomes. Another promising area of interest is the integration of intracoronary imaging modalities such as IVUS and optical coherence tomography (OCT) [61]. Imaging-guided DCB angioplasty has shown initial promising results by optimizing lesion preparation, minimizing geographical miss, and potentially enhancing clinical outcomes. Accurate segment targeting and the prevention of geographic miss require the acquisition of matched angiographic projections before and after the procedure, supported by anatomical landmarks such as side branches. According to the DCB-ARC definition [62], procedural success following DCB angioplasty is achieved when residual stenosis is <40% (preferably <30%), TIMI 3 flow is preserved, and no major dissections are present—ideally confirmed by quantitative coronary angiography. The consortium also recommends a 4-week DAPT regimen for patients undergoing DCB-only PCI in stable coronary settings. Nevertheless, clinical evidence remains limited for alternative antiplatelet strategies, including early de-escalation or P2Y12 inhibitor monotherapy [62]. Despite adequate lesion preparation, bailout stenting remains an integral component of DCB strategies when suboptimal angiographic results occur. In the REC-CAGEFREE I trial, 9.4% of patients initially treated with DCBs required rescue stenting, predominantly due to type D–F dissections or residual stenosis >30% [63]. This figure aligns with recent large-scale data such as those from the EASTBOURNE registry, where bailout stenting was necessary for 7.8% of lesions treated with sirolimus-coated balloons [64]. Notably, predictors of bailout stenting include de novo lesions, smaller vessel diameter, and shorter inflation time [64]. Older studies had reported higher rates (20–28%), likely due to less standardized lesion preparation and lower tolerance for residual angiographic imperfections [65]. When bailout is needed, contemporary data support the use of drug-eluting stents over bare-metal stents, with no significant increase in adverse events such as target lesion revascularization or stent thrombosis at a mid-term follow-up [66]. These findings reinforce the importance of optimal lesion preparation and patient selection to preserve the benefits of a ‘leave nothing behind’ strategy [5], while confirming the safety of a provisional stenting approach when clinically indicated. Future randomized trials systematically incorporating advanced imaging guidance in DCB strategies could significantly refine patient selection and procedural success. In conclusion, DCB angioplasty provides a compelling treatment strategy in contemporary interventional cardiology practice, characterized by favorable clinical outcomes, particularly when the avoidance of long-term metallic implants is advantageous. Nevertheless, to ensure wider acceptance and precise clinical positioning of DCB therapy, dedicated research with long-term outcomes and refined patient selection criteria remains imperative. Ultimately, continued technological advancements, well-designed clinical trials, and evidence-based refinements in interventional techniques will consolidate the clinical utility and therapeutic potential of drug-coated balloons in coronary artery disease management.

## Data Availability

No new data were created or analyzed in this study. Data sharing is not applicable to this article.

## Abbreviations

The following abbreviations are used in this manuscript:

ACS	Acute Coronary Syndromes
BMS	Bare-Metal Stent
CAD	Coronary Artery Disease
CTO	Chronic Total Occlusion
DAPT	Dual Antiplatelet Therapy
DCB	Drug-Coated Balloon
DEB	Drug-Eluting Balloon
DES	Drug-Eluting Stent
DM	Diabetes Mellitus
HBR	High Bleeding Risk
ISR	In-Stent Restenosis
IVUS	Intravascular Ultrasound
LLL	Late Lumen Loss
MACE	Major Adverse Cardiovascular Event
NSTEMI	Non-ST Elevated Myocardial Infarction
NCB	Non-compliant Balloon
OCT	Optical Coherence Tomography
PCB	Paclitaxel-Coated Balloon
PCI	Percutaneous Coronary Intervention
RCT	Randomized Controlled Trial
SAPT	Single Antiplatelet Therapy
SCB	Sirolimus-Coated Balloon
STEMI	ST-Elevated Myocardial Infarction
SVD	Small-Vessel Disease
TLF	Target Lesion Failure
TLR	Target Lesion Revascularization
TVR	Target Vessel Revascularization
